# A preclinical investigation into the potential associations of geraniin with ulcerative colitis alleviation through integrated multi-omics and *in vivo* analysis

**DOI:** 10.3389/fmed.2026.1821762

**Published:** 2026-05-13

**Authors:** Chang Cheng, Wei Wang, Tingting Zheng, Xiangmin Shi, Chong Fu

**Affiliations:** 1 Department of Gastroenterology, Anqing Municipal Hospital, Anqing, Anhui, China; 2 School of Life Sciences, Anqing Normal University, Anqing, Anhui, China

**Keywords:** geraniin, macrophage polarization, neutrophil extracellular traps, neutrophil infiltration, ulcerative colitis

## Abstract

**Objective:**

This study aims to investigate the potential molecular mechanisms and alleviating effects of geraniin, a natural polyphenol compound, within a murine model of ulcerative colitis (UC), thereby providing preclinical insights that may inform future translational strategies.

**Methods:**

Potential targets of geraniin for UC were identified by screening databases such as the Comparative Toxicogenomics Database and SwissTargetPrediction. UC transcriptomic and single-cell data were sourced from GEO (GSE47908, GSE214695). Differential gene analysis used limma, co-expression modules via WGCNA, enrichment with ClusterProfiler for GO/KEGG, and immune cells via CIBERSORT. Single-cell analysis employed Seurat and AUCell to identify targeted subpopulations. *In vivo*, DSS-induced UC mice were grouped as control, model, and model + geraniin (30, 60 mg/kg). Effects were assessed by DAI, histopathology, Western blot, and immunofluorescence.

**Results:**

Geraniin targeted 27 genes, which were integrated with 337 UC-related genes to construct a protein–protein interaction network. MCODE analysis identified a key subnetwork comprising 42 genes. GO and KEGG analyses suggested that the potential effects of geraniin may be linked to inflammatory pathways such as IL-17, TNF, and CXCR chemokine signaling. In the single-cell dataset GSE214695, AUCell scores indicated an enrichment of drug targets in macrophage and neutrophil clusters. In this murine model, *in vivo* experiments indicated that geraniin administration was associated with reduced DAI scores, improved colon length, and the alleviation of mucosal damage and inflammatory cell infiltration. Immunofluorescence analysis revealed that geraniin treatment was associated with a reduced presence of markers for M1 macrophages (F4/80+/CD86+), neutrophils, and NET formation (CitH3+/MPO+), while correlating with an increase in M2 macrophages markers (F4/80+/CD206+). Western blot analysis showed that geraniin treatment correlated with the downregulation of NOS2 and upregulation of PPARG expression, which may contribute to mitigating the inflammatory response observed in this model.

**Conclusion:**

These findings suggest that geraniin is associated with the alleviation of DSS-induced UC in mice, potentially through the modulation of phenotypic markers related to macrophages and neutrophils.

## 1 Introduction

Inflammatory bowel disease (IBD) is triggered by intestinal inflammation and epithelial damage, classified as chronic gastrointestinal inflammation, with UC and CD as its primary forms, UC being particularly prevalent, marked by nonspecific, refractory, and recurrent traits (1). UC adversely affects patients’ quality of life, and in severe instances, it can gradually progress into colon cancer, jeopardizing patients’ lives (2). Statistics reveal (3) that IBD impacts approximately 20 million individuals globally, constituting 0.3% of the total population. According to recent research, the incidence of IBD is increasing worldwide, particularly in Asia. In China, the incidence of IBD has markedly risen, and it is projected that by 2025, the number of patients will reach 1.5 million. Furthermore, studies indicate that the peak incidence age for IBD is 15–25 years, suggesting a shift toward a younger demographic. Presently, drug treatment regimens such as glucocorticoids and immunosuppressants are frequently employed in clinical practice for UC, but they are limited by a high recurrence risk and numerous side effects, making the exploration of superior treatment options crucial (4).

Geraniin, a natural polyphenolic compound, is prevalent in common plants such as geranium, longan, and *Phyllanthus urinaria*, with raw materials that are readily available and sustainably cultivable. It exhibits favorable water solubility and stability, is purifiable through conventional solvent extraction combined with column chromatography, dispensing with the need for extreme conditions, thus amenable to large-scale production. Furthermore, its molecular structure is rich in phenolic hydroxyl groups, particularly the hexahydroxydiphenoyl (HHDP) and dehydrohexahydroxydiphenoyl (DHHDP) units. These groups not only confer upon it a robust antioxidant capacity, efficiently eliminating lipid peroxides (LPO), but also impede the progression of free radical condensation reactions due to pronounced steric hindrance. Analysis via UHPLC-ESI-Q-TOF-MS/MS demonstrated that no free radical chelation products were detected in geraniin either *in vivo* or *in vitro*, indicating that upon entering human cells, it is less likely to cross-link with biomolecules or their free radical intermediates, thereby mitigating the risk of inducing mutations or carcinogenesis. This attribute renders it a safe and efficient natural inhibitor of ferroptosis (5). Moreover, geraniin suppresses inflammatory signaling pathways such as NF-κB and MAPK, diminishing the release of pro-inflammatory factors like TNF-α and IL-6, thus exhibiting potential therapeutic efficacy against inflammatory diseases such as arthritis (6, 7).

Leveraging its merits of low production cost, multi-target synergistic effects, and minimal toxicity, geraniin demonstrates preclinical potential for addressing inflammation-related diseases such as UC. However, extant literature on the specific anti-inflammatory mechanism of geraniin in the context of intestinal inflammation is scarce. Therefore, to investigate the potential molecular associations underlying the effects of geraniin in ulcerative colitis, this study integrates network pharmacology, transcriptomics, and single-cell sequencing technologies. These bioinformatic insights were subsequently correlated with observations from murine experiments, thereby establishing a preclinical basis for further exploring its potential translational value in ulcerative colitis.

## 2 Method

### 2.1 Sources of data

The chemical-genetic interaction data for geraniol derivatives are elegantly sourced from the Comparative Toxicogenomics Database, SwissTargetPrediction, and the Binding DB database. Transcriptional datasets pertinent to active ulcerative colitis (UC) were meticulously acquired from the GEO database,^1^ encompassing the GSE47908 dataset, which includes samples from 45 UC patients juxtaposed against 15 healthy subjects, and the single-cell sequencing dataset GSE214695, comprising specimens from 6 UC patients and an equal number of healthy individuals.

### 2.2 Differential gene analysis

For the GSE47908 dataset, a sophisticated differential gene analysis was conducted, employing the “limma R” package to discerningly filter differentially expressed genes (DEGs). The results were artfully visualized using the “ggplot2” R package. The criteria established for identifying DEGs were stringent, requiring a Log2│FC│ ≥ 2 and Adjusted *p*-value ≤ 0.05.

### 2.3 Weighted gene co-expression network analysis

Utilizing the WGCNA R package, an intricate co-expression network was constructed. The soft threshold was precisely estimated with the aid of the pickSoftThreshold function. Subsequently, this threshold facilitated the creation of a correlation matrix, followed by the detailed computation of a topological overlap matrix (TOM). Hierarchical clustering was then performed using the hclust function, and genes were grouped based on topological overlap dissimilarity (1-TOM). The dynamic tree cut algorithm was employed to delineate gene modules. Module membership (MM), reflecting the correlation between gene expression levels and module eigengenes, was calculated to ascertain the significance of genes within their respective modules. Elevated MM values signify a profound correlation with module eigengenes, indicating that these genes are pivotal within their modules.

### 2.4 Pathway enrichment analysis

Utilizing the ClusterProfiler package within the R software environment, we conducted Gene Ontology (GO) functional enrichment and Kyoto Encyclopedia of Genes and Genomes (KEGG) pathway enrichment analyses1 upon the identified common target genes. The GO analysis encompassed three principal domains: biological process (BP), cellular component (CC), and molecular function (MF). Entries exhibiting statistical significance (*p* < 0.05) were selected, and their visualization was achieved through bar plots generated via the ggplot2 package.

### 2.5 Immune signature characterization

Employing the CIBERSORT algorithm implemented in R, we computed the relative proportions of various immune cell populations within each sample of the dataset. Subsequent visualization through box plots lucidly revealed significant disparities in immune cell infiltration profiles between normal control samples and those derived from individuals afflicted with ulcerative colitis. Furthermore, correlation bubble plots were generated to vividly illustrate the intricate interrelationships between the core target genes and specific immune cell subsets.

### 2.6 Identification of the pivotal molecular network governed by geraniin in UC

We systematically delineated the intersecting targets implicated in both geraniin’s activity and the pathophysiology of ulcerative colitis using the VennDiagram package. Based on these common targets, a high-confidence protein–protein interaction (PPI) network was constructed leveraging the STRING 11.5 database. Through the synergistic application of Cytoscape’s visualization capabilities and the MCODE algorithm for module detection, we performed an in-depth interrogation of the interaction modules, thereby unveiling the critical molecular network and associated hub gene clusters through which geraniin exerts its regulatory influence on ulcerative colitis.

### 2.7 Geraniin’s effects on cellular subpopulations in UC

Initially, we undertook a re-analysis of single-cell RNA sequencing (scRNA-seq) data originating from the colonic tissues of patients diagnosed with ulcerative colitis. Rigorous quality control measures were applied to filter out cells characterized by fewer than 200 or exceeding 8,000 Unique Molecular Identifiers (UMIs), as well as those displaying a mitochondrial gene fraction surpassing 20%. The analytical workflow, orchestrated using the “Seurat” package (version 5.1.0^2^), encompassed data normalization, identification of highly variable features, data scaling, principal component analysis (PCA), dimensionality reduction via Uniform Manifold Approximation and Projection (UMAP), and graph-based unsupervised clustering. Mitigation of batch effects was accomplished using the “Harmony” algorithm (version 0.1). Differentially expressed genes (DEGs) defining each cluster were identified, and canonical marker genes for these clusters were visualized employing “scRNAtoolVis” (version 0.1.9), predicated upon an absolute log2 fold change threshold of 1.5 and an adjusted *p*-value cutoff of 0.01. Subsequent comparative analyses pinpointed DEGs distinguishing specific clusters of interest. Cell type identities were assigned based on established canonical markers. Moreover, the AUCell software (version 1.26.0) was implemented to quantitatively assess the enrichment of putative drug target gene signatures within the distinct cellular aggregates.

### 2.8 Experimental animals

All animal experiments were conducted in strict accordance with protocols reviewed and sanctioned by the Anqing Normal University Animal Care and Experimentation Committee (Anhui, China, No. AQNU2024037). All experiments were conducted in accordance with the guidelines of Animal Research and the study adhered to the ARRIVE guidelines ensuring compliance with ethical standards for animal research.

Twenty wild-type, specific pathogen-free (SPF) grade, eight-week-old male C57BL/6 mice, weighing between 20 and 25 grams, were procured from Shanghai SLAC Laboratory Animal Co., Ltd. All animals were housed within the SPF-grade vivarium at Anqing Normal University under controlled environmental conditions, maintaining a room temperature of 20–23 °C, a 12-h light/dark cycle, and relative humidity between 40 and 60%. Prior to the initiation of experimental procedures, the mice underwent a one-week acclimatization period, during which they had ad libitum access to standard laboratory chow and water.

### 2.9 Mouse grouping and model induction

The mice were randomly allocated into four experimental cohorts (*n* = 5 per cohort) using a computer-generated random number sequence: a control group, a DSS-induced colitis group (Model group), a DSS + Geraniin (30 mg/kg) group (Model + GE30mg/kg), and a DSS + Geraniin (60 mg/kg) group (Model + GE 60 mg/kg). Excluding the Control group, mice in the remaining three cohorts received drinking water supplemented with 2.5% (w/v) DSS for seven consecutive days to induce experimental colitis. Concurrently, all designated treatments (vehicle or geraniin) were administered once daily via oral gavage over the same seven-day period. The selection of geraniin dosages (30 and 60 mg/kg) was determined based on its established pharmacokinetic profiles and recent preclinical studies demonstrating its anti-inflammatory, mucosal protective efficacy, and tolerability in DSS-induced murine colitis models, as well as other *in vivo* inflammatory conditions within similar dosage ranges (8). The Disease Activity Index (DAI) was assessed daily according to the criteria delineated by Cooper et al. (9), incorporating evaluations of: weight loss (0, none, 1: 1–5%; 2: 6–10%; 3: 11–15%; 4: >15%), stool consistency (0, normal, well-formed pellets, 2, loose stools, pasty/semi-formed, not adhering to the anus; 4: diarrhea, liquid stools adhering to the anus), and fecal blood presence (0, negative occult blood or no visible blood, 2, positive occult blood; 4: gross rectal bleeding). The composite score from these three parameters was divided by three to obtain the final DAI value. To explicitly minimize observer bias, the daily DAI scoring and subsequent macroscopic evaluations were conducted by an independent investigator who was strictly blinded to the experimental group allocations. During the seven-day experimental period, no anesthesia was administered as the oral gavage and clinical assessments were non-invasive and performed to minimize animal distress. At the end of the study, euthanasia was administered via CO_2_ inhalation with a chamber displacement rate of 30% per minute, a procedure carried out in accordance with the American Veterinary Medical Association (AVMA) Guidelines for the Euthanasia of Animals. Death was confirmed by the cessation of heartbeat and respiration prior to tissue collection. The entire colon was then carefully excised from all animals in each group (*n* = 5) and thoroughly lavaged with phosphate-buffered saline. To ensure data accuracy and minimize potential bias from post-mortem tissue changes, the absolute colon length was measured immediately after dissection before any further processing. Subsequently, colonic tissues were harvested for subsequent analyses.

### 2.10 Histopathological assessment

A 0.5 cm segment of tissue, consistently obtained from the distal colon of each mouse, was fixed in 4% paraformaldehyde. These fixed tissues were then processed through standard paraffin embedding procedures and sectioned. Tissue sections were stained with Hematoxylin and Eosin (H&E) and subsequently examined via light microscopy. Histopathological damage was quantitatively assessed based on established scoring criteria (10), evaluating the severity of crypt architectural disruption, the depth and extent of mucosal lesions, and the degree of inflammatory cell infiltration. The summation of scores from these individual components constituted the overall histopathological injury score.

### 2.11 Western blotting analysis

Approximately 100 mg of minced colonic tissue per sample was homogenized in 150 μL of ice-cold RIPA lysis buffer and incubated on ice for 30 min to facilitate protein extraction. Total protein concentrations were determined using a Bicinchoninic Acid (BCA) protein assay kit. Equal amounts of protein lysate were resolved by 10% sodium dodecyl sulfate-polyacrylamide gel electrophoresis (SDS-PAGE) and subsequently transferred onto polyvinylidene difluoride (PVDF) membranes.1 Membranes were blocked for 2 h at room temperature with 5% non-fat dry milk dissolved in Tris-buffered saline containing 0.1% Tween-20 (TBST) under constant agitation. Following blocking, membranes were incubated overnight at 4 °C with primary antibodies directed against GAPDH (1:10000), PPARG (1:5000), and NOS2 (1:5000). After incubation, membranes were washed thrice with TBST for 10 min each. Subsequently, they were incubated with the corresponding horseradish peroxidase (HRP)-conjugated secondary antibody (1:10000) for 2 h at room temperature with agitation. Following three further 10-min washes in TBST, immunoreactive bands were visualized using an enhanced chemiluminescence (ECL) substrate detection system and imaged with a gel documentation apparatus. Band intensities were quantified using ImageJ software through densitometric analysis.

### 2.12 Immunofluorescence staining

Immunofluorescence staining was conducted following established protocols using primary antibodies targeting Ly-6G (Bio-Rad, MCA771GA), Citrullinated Histone H3 (CitH3; NOVUS, NB100-57135), Myeloperoxidase (MPO; ABCAM, AB208670), F4/80 (Proteintech, 28463-1-AP), CD86 (BIOSS, bs-1035R), and CD206 (Santa Cruz Biotechnology, SC-58986). Tissue sections were incubated with these primary antibodies overnight at 4 °C. After thorough rinsing with PBS, sections were incubated with appropriately labeled fluorescent secondary antibodies for 1 h at 37 °C in a light-protected environment. Nuclear counterstaining was performed using 4′,6-diamidino-2-phenylindole (DAPI). Finally, the stained sections were mounted and examined using a laser scanning confocal microscope for image acquisition and analysis. The Mean Fluorescence Intensity of specific markers and the percentages of positive cells were quantified in a blinded manner using ImageJ software.

### 2.13 Statistical analysis

All statistical computations were performed utilizing GraphPad Prism software (version 9.5.1). Quantitative data are expressed as the mean ± standard deviation (S.D.). For comparative analyses involving multiple groups, the Kruskal-Wallis test was employed for data sets not adhering to a normal distribution, whereas normally distributed data were analyzed using one-way analysis of variance (ANOVA) followed by Tukey’s honestly significant difference *post-hoc* test for multiple comparisons. A *p*-value threshold of less than 0.05 was deemed statistically significant across all analyses.

## 3 Results

### 3.1 Weighted gene co-expression network analysis

**Figure 1:**
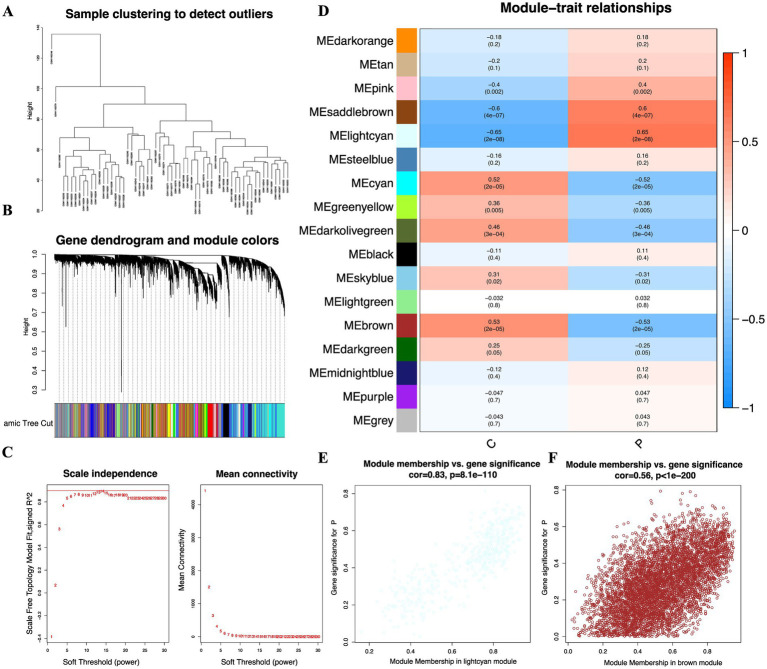
WGCNA identifying key gene modules associated with UC in the GSE47908 dataset. **(A)** Sample clustering dendrogram of 60 samples to detect and exclude potential outliers. **(B)** Gene dendrogram obtained by average linkage hierarchical clustering. The colored rows below the dendrogram represent the 17 distinct gene co-expression modules identified by the dynamic tree cut algorithm. **(C)** Analysis of network topology to determine the optimal soft-thresholding power. A soft threshold of β = 14 was selected to ensure a scale-free network (*R*^2^ > 0.9). **(D)** Heatmap of module-trait relationships illustrating the correlation between the 17 gene modules and disease phenotypes. **(E,F)** Scatter plots depicting the highly significant correlation between GS and MM for genes within the lightcyan **(E)** and brown **(F)** modules, respectively.

Microarray data were procured from the GSE47908 dataset. Following preprocessing, an expression matrix encompassing 20,021 genes across 60 samples was meticulously constructed. To forge gene co-expression modules, the top 5,000 genes exhibiting the highest average expression were selected. Outlier detection revealed no significant anomalies within the dataset. Hierarchical clustering was employed to group the samples (Figure 1A). To construct gene modules for subsequent correlation with clinical phenotypes, soft threshold powers ranging from 1 to 30 were evaluated. A soft threshold of 14 was adopted (Figure 1C), ensuring an *R*^2^ value exceeding 0.9 and reaffirming the network’s scale-free topology. Subsequently, a hierarchical clustering dendrogram was constructed based on the topological overlap matrix, yielding 17 distinct modules (Figure 1B). Notably, the lightcyan and brown modules exhibited the most robust association with ulcerative colitis (Figure 1D). Scatter plots further underscored a pronounced correlation between GS and MM within these modules (Figures 1E,F). Thus, the lightcyan and brown modules are posited as pivotal in the context of ulcerative colitis.

### 3.2 Differential gene analysis

**Figure 2:**
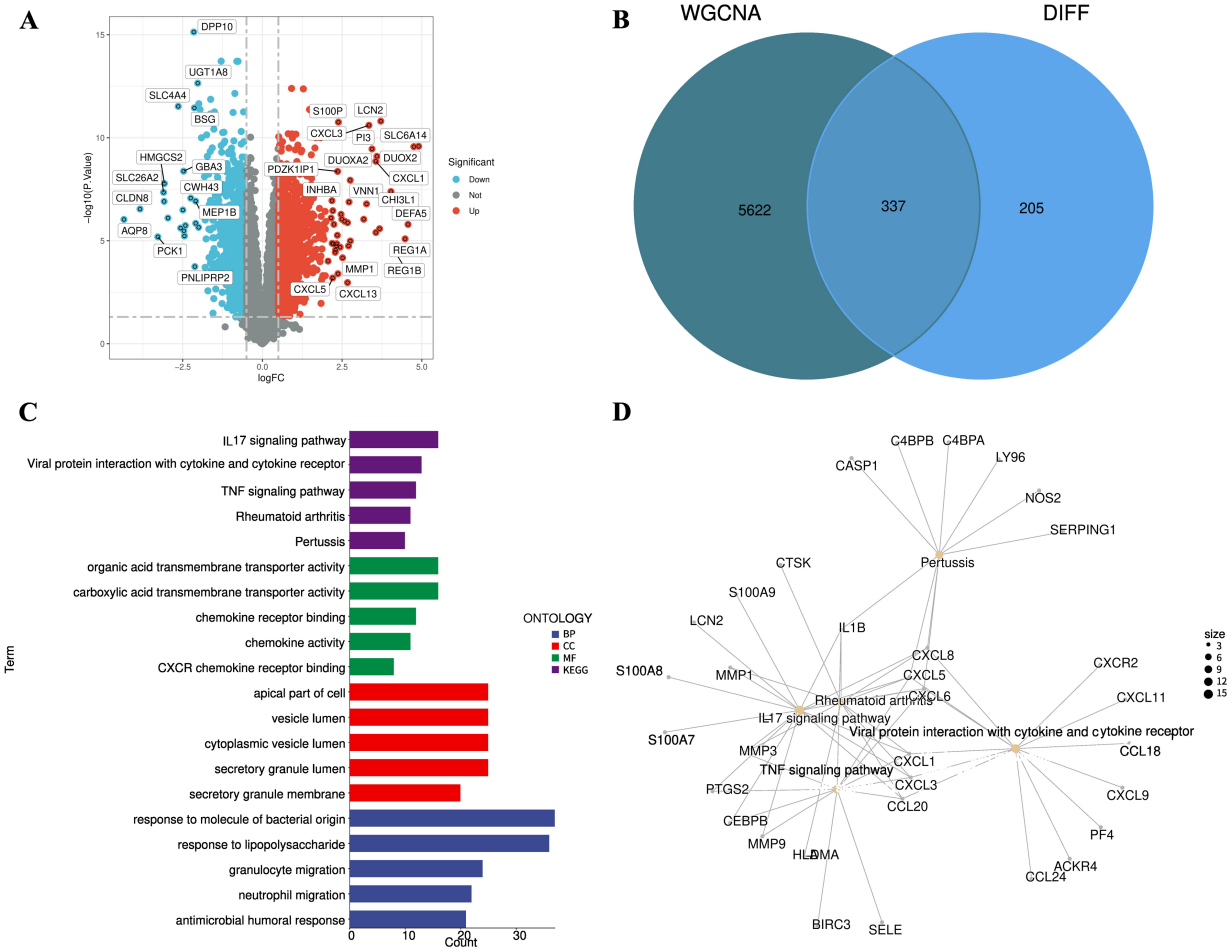
Identification and functional enrichment analysis of disease-associated genes in UC. **(A)** Volcano plot displaying the expression patterns of 542 DEGs between UC and normal intestinal tissues from the GSE47908 dataset. Red and blue dots represent significantly up-regulated (*n* = 326) and down-regulated (*n* = 216) genes, respectively. **(B)** Venn diagram identifying 337 intersecting disease-associated genes shared between the identified DEGs and the key WGCNA modules (lightcyan and brown). **(C)** Bar plot illustrating the top significantly enriched GO terms and KEGG pathways for the 337 disease-associated genes. **(D)** cnetplot depicting the complex molecular linkages between the significantly enriched pathways and their corresponding genes.

Leveraging the GSE47908 dataset, differential expression analysis via the limma package unveiled 542 genes with divergent expression profiles between ulcerative colitis and normal intestinal tissues. Of these, 326 genes were upregulated, while 216 were downregulated (Supplementary Table S1). A volcano plot, rendered using R, highlights the 20 genes with the most substantial expression alterations in the ulcerative colitis cohort (Figure 2A). These expression shifts may be intricately linked to the genetic predisposition to ulcerative colitis.

### 3.3 Pathway enrichment analysis

WGCNA analysis facilitated the comparison of genes within the lightcyan and brown modules against differentially expressed genes, culminating in the identification of 337 disease-associated genes (Figure 2B). GO enrichment analysis revealed pathways predominantly engaged in responses to bacterial molecules and lipopolysaccharides, as well as components like secretory granule and cytoplasmic vesicle lumens, alongside functions such as CXCR chemokine receptor binding and chemokine activity. KEGG enrichment underscored pathways including IL-17 signaling, viral protein interactions with cytokines and their receptors, rheumatoid arthritis, pertussis, and TNF signaling (Figures 2C,D).

### 3.4 Geraniin modulates key networks and immune features in UC

**Figure 3:**
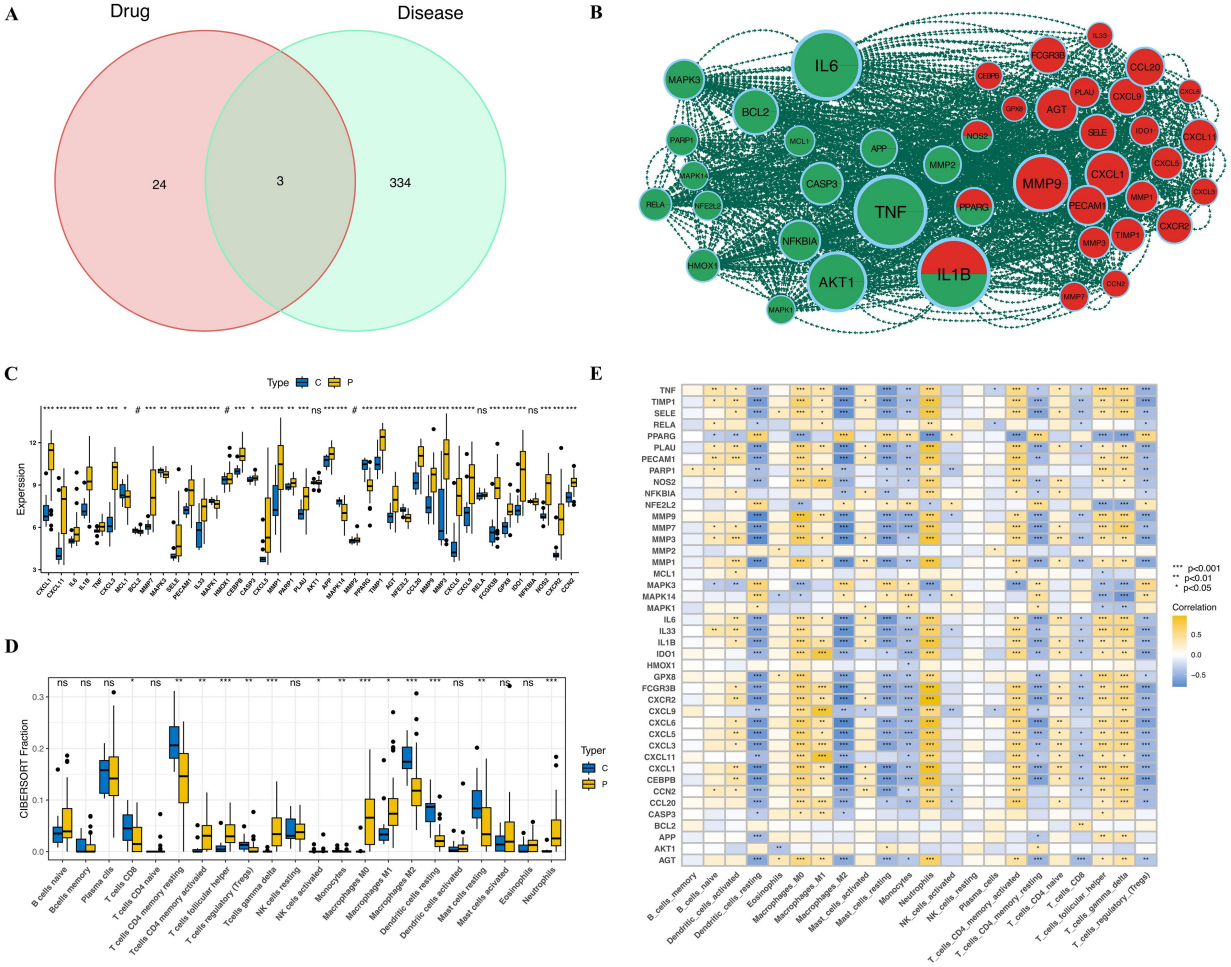
Identification of geraniin targets and immunological features in the UC microenvironment. **(A)** Venn diagram illustrating the intersection between 27 geraniin target genes and the 337 UC-associated genes. **(B)** PPI network diagram constructed via the STRING database. A key subnetwork of 42 hub genes was extracted using the MCODE algorithm in Cytoscape. **(C)** Box plot showing the comparative expression levels of the key subnetwork genes in normal versus diseased tissues. **(D)** Box plot comparing the relative infiltration levels of 22 distinct immune cell types between healthy controls and UC patients, evaluated using the CIBERSORT algorithm. **(E)** Correlation heatmap examining the complex relationship between the enrichment scores of specific immune cells and the expression levels of the key subnetwork genes.

Through an exhaustive investigation employing the sophisticated resources of the SwissTargetPrediction, CTD, and BindingDB databases, we have discerned 27 target genes intricately linked to geraniin (Supplementary Table S2). Notably, PPARG, NOS2, and IL1B emerged as shared targets for both ulcerative colitis and geraniin (Figure 3A). To craft a refined protein–protein interaction (PPI) network, we integrated 337 disease-associated genes and molecular drug targets into the STRING online database. Following the exclusion of aberrant proteins, an elegant network encompassing 298 proteins was unveiled. By harnessing the MCODE algorithm within Cytoscape, we illuminated 42 cardinal subgroup genes, achieving an impressive score of 26.732 (Figure 3B). As depicted in the meticulously crafted box plot, pronounced disparities in these pivotal subgroup genes were evident between the disease and control cohorts (Figure 3C). Moreover, the box plot and immune heatmap eloquently reveal that the principal regulatory targets of these subgroup genes predominantly reside within the immunological pathways of M1 macrophages, neutrophils, and activated CD4 T cells (Figures 3D,E), underscoring their profound role in orchestrating immune responses in ulcerative colitis.

### 3.5 Geraniin’s effects on cellular subpopulations in UC

In both healthy control (HC) and ulcerative colitis (UC) tissues (*n* = 6 per group), a striking positive correlation was observed between quantified gene expression (nCount_RNA) and the number of detected genes per cell (nFeature_RNA) (Supplementary Figure S1A). To ensure the reliability of downstream analyses, rigorous quality control (QC) criteria were applied. Specifically, we meticulously excluded low-quality cells exhibiting gene counts surpassing 8,000 or falling below 250, alongside those with mitochondrial gene proportions exceeding 20%. The distributions of these key QC metrics across all 12 samples before and after filtration are clearly delineated in Supplementary Figures S1B,C. Following QC, principal component analysis (PCA) was performed to reduce dimensionality, yielding 20 principal components for subsequent clustering (Supplementary Figure S1D).

**Figure 4:**
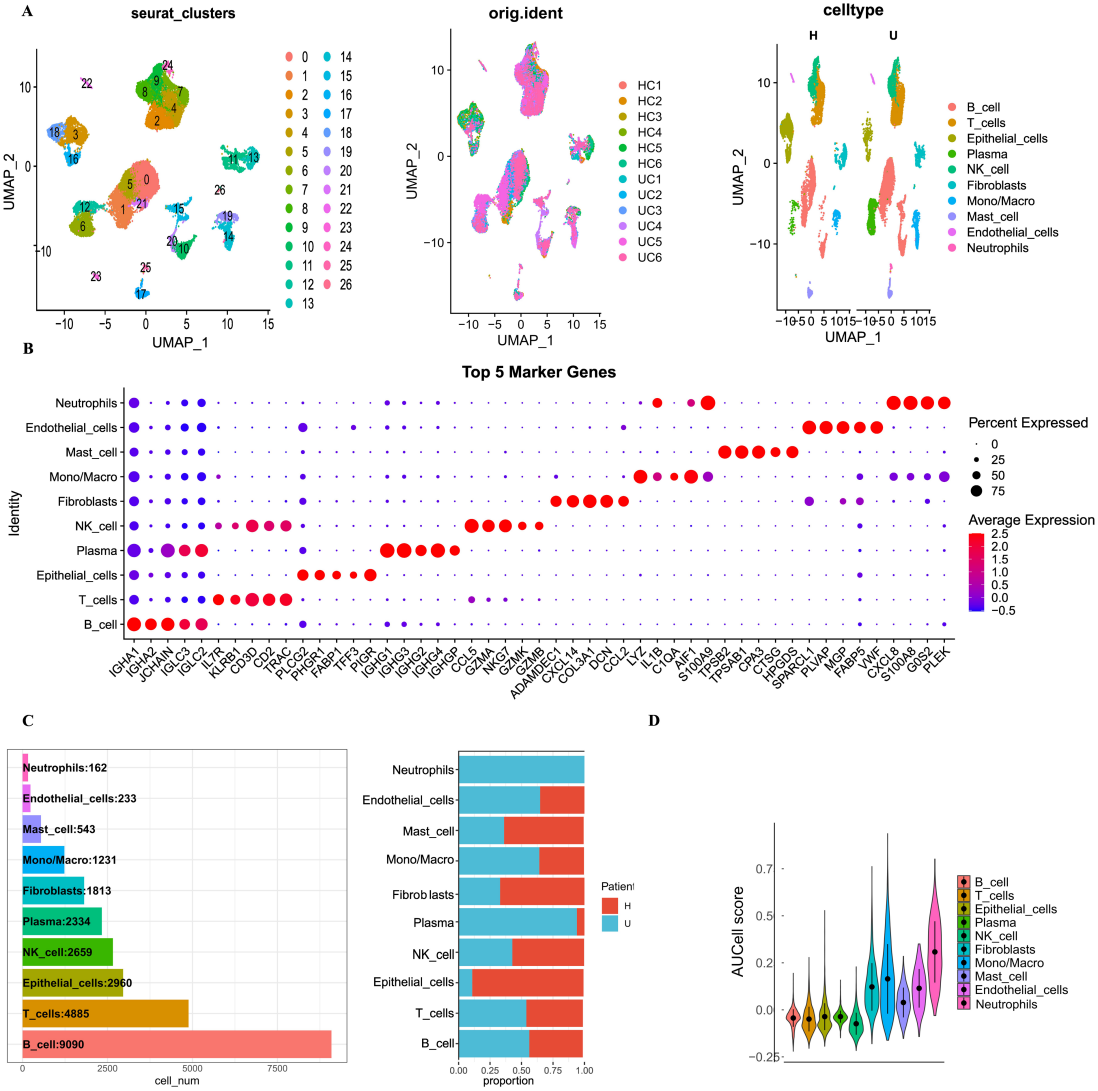
Single-cell transcriptomic profiling and identification of Geraniin-targeted cellular subpopulations. **(A)** UMAP plots displaying the global distribution of single cells after batch-effect correction via Harmony. Cells are color-coded by the 27 initial Seurat subclusters (left), sample origin (middle), and the 10 final merged and annotated major cell types (right). **(B)** Bubble plot validating the accuracy of cell annotation. It represents the expression levels and percentage of expressing cells for the top 5 algorithmically derived marker genescorresponding to each of the 10 annotated cell subpopulations. **(C)** Stacked bar plots detailing the absolute cell counts (left) and relative proportions (right) of each cell type across different samples. **(D)** Violin plots showing the comparative analysis of AUCell enrichment scores across the identified cell subpopulations, demonstrating that geraniin’s specific target gene signatures are predominantly enriched within the macrophage and neutrophil clusters.

Following unsupervised clustering, the single cells were initially partitioned into 27 distinct Seurat subclusters (clusters 0–26). To accurately determine the cellular identity of each subcluster, we analyzed the expression profiles of established canonical marker genes across all 27 clusters, as comprehensively visualized in Supplementary Figure S2. Based on these distinct expression patterns, the 27 subclusters were confidently annotated and subsequently merged into 10 major cell types: neutrophils, endothelial cells, mast cells, monocytes/macrophages, fibroblasts, plasma cells, NK cells, epithelial cells, T cells, and B cells. The distribution of these 10 cell types and their sample origins was elegantly visualized on UMAP plots (Figure 4A), and their quantitative proportions across all samples were detailed in Figure 4C. To robustly validate the accuracy of our cell type annotations, the FindAllMarkers function was subsequently employed to identify the top defining marker genes for each of the 10 major subpopulations. The expression of the top five data-driven marker genes for each group was portrayed through a dot plot (Figure 4B). Notably, these unbiased, algorithmically derived top genes perfectly matched the canonical markers for their respective cell lineages, thereby flawlessly confirming the accuracy of our initial annotations. Ultimately, the cellular distribution of drug target AUCell function scores eloquently demonstrated that geraniin predominantly exerts its influence upon macrophages and neutrophils (Figure 4D).

### 3.6 Geraniin reduces severity in UC

**Figure 5:**
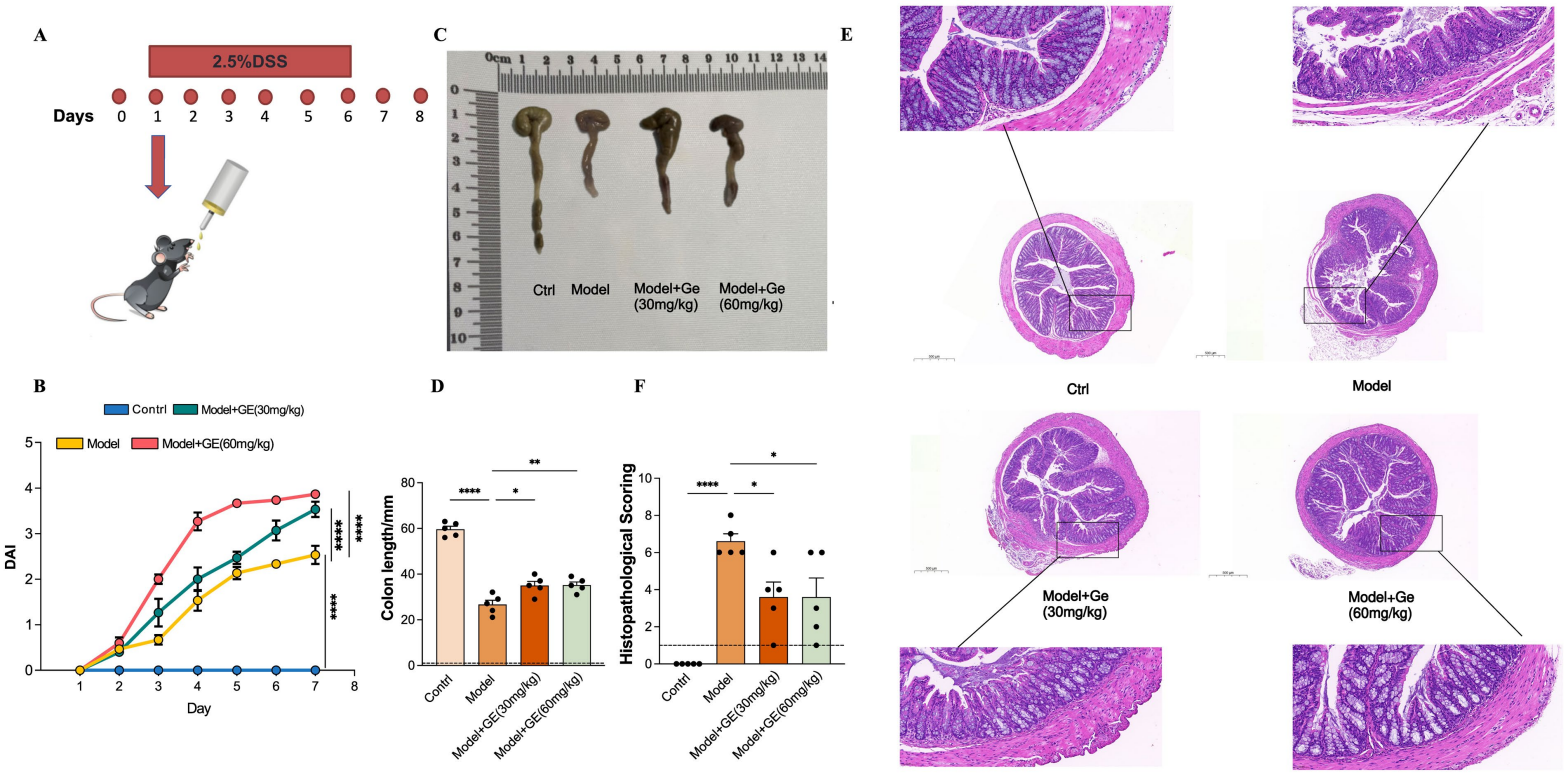
Geraniin alleviates disease severity and colonic histopathology in DSS-induced ulcerative colitis. **(A)** Schematic representation of the experimental protocol for inducing chronic colitis in C57BL/6 mice using DSS. **(B)** Line graph with error bars illustrating the DAIscores over time for mice in the control, model, model + 30 mg/kg geraniin, and model + 60 mg/kg geraniin groups. **(C)** Representative macroscopic images of colons from each experimental group. **(D)** Bar chart quantifying the absolute colon lengths across the four groups. **(E)** Representative H&E stained images of colonic sections evaluating mucosal damage and inflammatory cell infiltration. **(F)** Bar chart quantifying the colonic histopathological scores. Data are presented as mean ± standard deviation (SD). Statistical significance across multiple groups was determined using one-way ANOVA followed by Tukey’s *post-hoc* test. *p < 0.05, **p < 0.01, ***p < 0.001, ****p < 0.0001.

To explore the potential protective effects of geraniin against colitis, an experimental model was established through the administration of 2.5% DSS (Figure 5A). The findings revealed that mice exposed solely to DSS manifested significantly elevated DAI scores relative to the untreated control cohort (Figure 5B). Conversely, concurrent administration of geraniin was associated with a marked decrease in DAI scores, indicating a potential therapeutic benefit (Figure 5B). Furthermore, DSS induction led to a pronounced reduction in colon length compared to control animals; this pathological shortening was significantly ameliorated in mice receiving geraniin treatment (Figures 5C,D). Collectively, these data suggest that geraniin administration correlates with the mitigation of adverse clinical manifestations associated with DSS-induced ulcerative colitis in this murine model.

### 3.7 Effect of geraniin on colonic histopathology in UC

To further evaluate the potential protective associations of geraniin in the context of ulcerative colitis, comprehensive histological examinations of colonic tissues were performed. The analysis indicated that colonic sections from mice subjected to DSS induction exhibited severe pathological alterations, including pronounced submucosal edema, extensive infiltration by inflammatory leukocytes, and significant disruption of the crypt architecture. Notably, in mice concurrently treated with geraniin, we observed a significant reduction in the severity of crypt damage and epithelial injury. Consistently, geraniin administration was linked to a substantial decrease in the overall histological damage scores (Figures 5E,F). These observations suggest that geraniin potentially attenuates the intestinal tissue injury prompted by DSS administration in mice.

### 3.8 Geraniin diminishes immune cell infiltration in UC

**Figure 6:**
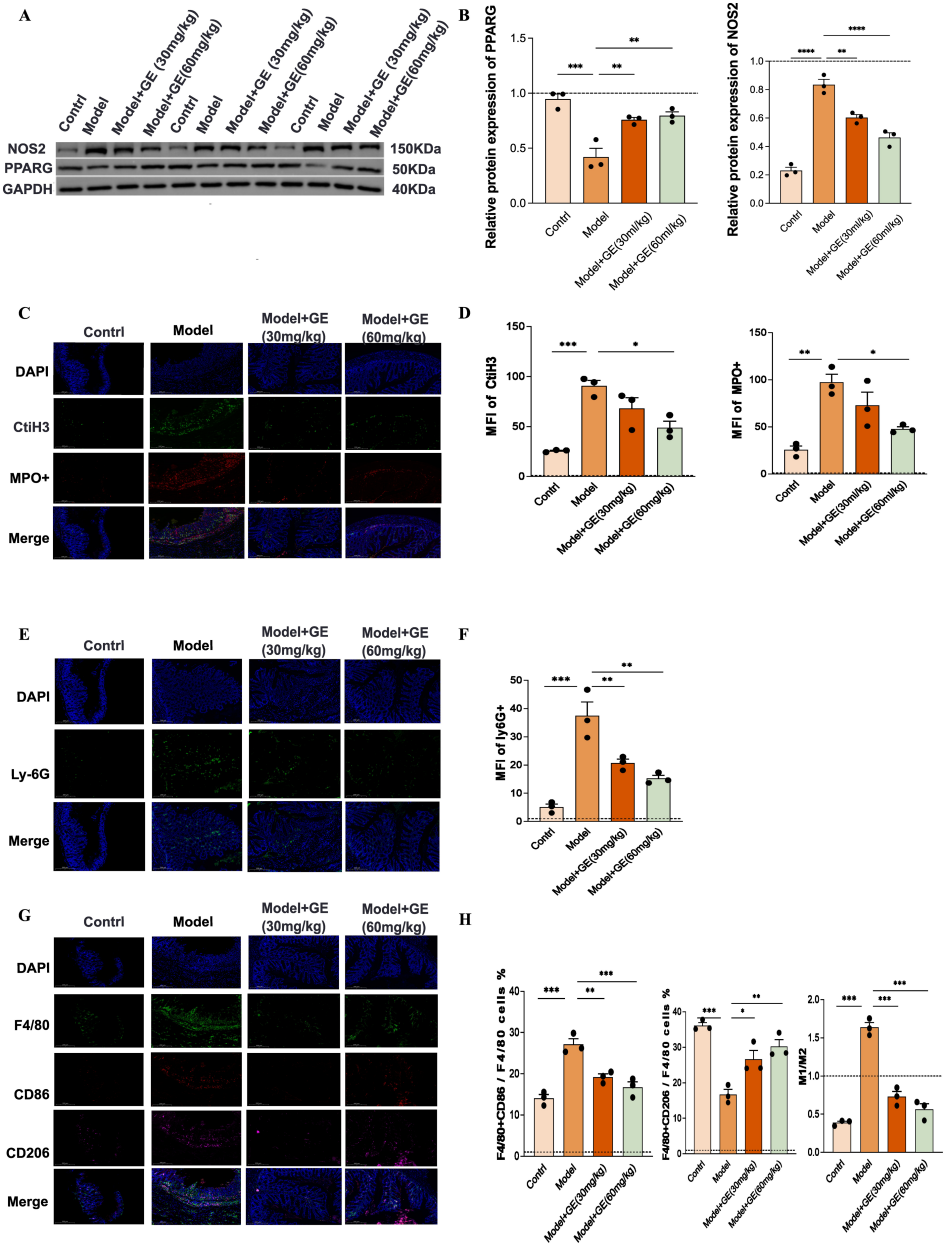
Effects of geraniin on targeted protein expression and phenotypic markers of neutrophils and macrophages in the colonic microenvironment. **(A)** Representative Western blot images depicting the protein expression levels of PPARG and NOS2 in colonic tissues across the control, DSS-induced model, and geraniin-treated (30 mg/kg and 60 mg/kg) groups. **(B)** Bar charts quantifying the relative densitometric protein expression levels of PPARG and NOS2. **(C)** Representative IF images evaluating NET formation markers, identified by the co-localization of CitH3 (green), MPO (red), and nuclear DAPI (blue) staining in colon tissues. **(D)** Bar charts quantifying the mean fluorescence intensity of CitH3 and MPO + signals. **(E)** Representative IF images assessing neutrophil infiltration markers in the colonic lamina propria, explicitly indicated by Ly-6G positivity (green). **(F)** Bar chart quantifying the MFI of Ly-6G + signals. **(G)** Representative IF images detecting macrophage polarization phenotypes in colon tissues. Pro-inflammatory M1 macrophages are identified by F4/80 (green) and CD86 (red) co-staining, while anti-inflammatory M2 macrophages are indicated by F4/80 (green) and CD206 (purple) co-staining. **(H)** Bar charts quantifying the percentages of F4/80 + CD86 + cells and F4/80 + CD206 + cells relative to total F4/80 + macrophages, alongside the calculated M1/M2 ratio. Data are presented as mean ± standard deviation (SD). Statistical significance across multiple groups was determined using one-way ANOVA followed by Tukey’s *post-hoc* test. *p < 0.05, **p < 0.01, ***p < 0.001, ****p < 0.0001.

To further elucidate the immunomodulatory effects of geraniin on inflammatory processes, we assessed the expression levels of critical molecular determinants. Our findings revealed that DSS induction led to a downregulation of PPARG and an upregulation of NOS2, whereas geraniin administration effectively reversed these aberrant protein expression levels (Figures 6A,B).

Given that the release of neutrophil extracellular traps (NETs) is a well-established mechanism by which neutrophils contribute to inflammatory pathology, we investigated NET formation via co-immunostaining for Myeloperoxidase (MPO) and Citrullinated Histone H3 (citH3). Compared to healthy controls, the colonic tissue from the DSS model group exhibited significantly elevated marker expression of NETs (MPO+/citH3+). Notably, geraniin treatment resulted in a marked reduction in these immunofluorescence markers associated with NET formation within the colonic tissue of these colitic animals (Figures 6C,D). Furthermore, consistent with our earlier bioinformatic predictions highlighting neutrophils and macrophages as key cellular targets of geraniin, we quantified neutrophil presence in the colonic tissue. Immunofluorescence analysis confirmed a substantial increase in the markers of neutrophils (Ly6G+) within the DSS group relative to controls. Geraniin administration, however, significantly reduced the immunofluorescence markers indicative of neutrophil infiltration in the colonic lamina propria (Figures 6E,F). Finally, we characterized macrophage subtypes within the colonic tissue sections. This analysis demonstrated a heightened prevalence of pro-inflammatory M1 macrophages (defined as F4/80+/CD86+) and a concomitant decrease in anti-inflammatory M2 macrophages (F4/80+/CD206+) in the colons of colitic mice compared to their control counterparts. Significantly, geraniin treatment was associated with a decreased presence of M1 macrophage markers and a concurrent increase in M2 macrophage markers within the colons of DSS-exposed mice (Figures 6G,H).

## 4 Discussion

Histological inflammation emerges as a cardinal peril for the recurrence of UC, necessitating refinements in therapeutic regimens and posing a significant risk of colorectal carcinoma. The infiltration and activation of neutrophils and macrophages within the intestinal mucosa constitute a key pathological mechanism associated with the progression of UC. Our inquiry suggests that geraniin is associated with the modulation of PPARG and NOS2 expression, which may contribute to the alleviation of intestinal inflammation observed in this model. Moreover, preliminary revelations from immunofluorescence assays suggest that geraniin treatment correlates with shifts in marker expression associated with pro-inflammatory M1 macrophages whilst concurrently increasing phenotypic markers of anti-inflammatory M2 macrophages. Simultaneously, the administration of geraniin was associated with a reduced incursion of neutrophils into lesion sites and an attenuation of phenotypic markers indicative of aberrant NET formation.

While previous literature has documented the general anti-inflammatory properties of geraniin, including its influence on macrophage polarization, these observations were predominantly derived from *in vitro* assays or models of other systemic diseases, such as cardiovascular disorders. To our knowledge, no prior studies have investigated whether geraniin is associated with the regulation of interplay of the macrophage-neutrophil axis within the *in vivo* microenvironment of ulcerative colitis. Crucially, although the involvement of macrophages and neutrophils is a well-established pathological feature of UC, our single-cell AUCell analysis provides a highly novel, drug-specific mechanistic insight. We found that the 27 putative target genes of geraniin are significantly and specifically enriched within these two immune cell subpopulations. This suggests that geraniin may not act as a broad, non-specific anti-inflammatory agent across the entire colonic tissue; rather, it potentially exerts its effects by modulating signaling networks localized within macrophages and neutrophils. When contextualized against other botanical therapeutics, geraniin exhibits a potentially more selective immunomodulatory profile. While well-characterized polyphenols, such as curcumin, resveratrol, and quercetin, typically exhibit broad-spectrum modulation across hundreds of targets and ubiquitous signaling pathways traversing multiple cell lineages (11), our AUCell data underscores a highly targeted mechanism for geraniin. This specific enrichment implies a more targeted pharmacological intervention with potentially reduced off-target impacts, contrasting sharply with the non-specific broad modulation characteristic of traditional polyphenols. Therefore, our study provides novel multi-omics and *in vivo* evidence suggesting that geraniin may orchestrate mucosal healing in UC by concurrently modulating markers of colonic macrophage polarization and reducing the marker expression associated with NET formation.

Macrophages, contingent upon the stimuli of their microenvironment, polarize into two disparate phenotypes: the classically activated (M1) macrophages, which, by secreting pro-inflammatory cytokines, inflict tissue damage and contribute to disease progression; and the alternatively activated (M2) macrophages, which release anti-inflammatory mediators. Importantly, recent paradigm shifts in mucosal immunology highlight the remarkable functional plasticity of intestinal macrophages. Rather than being terminally differentiated into fixed states, these cells exist along a dynamic continuum, capable of continuous phenotypic reprogramming in response to microenvironmental cues (12, 13). Within this highly plastic continuum, the classic M1 and M2 phenotypes evaluated in our study serve as critical diagnostic anchors representing the two functional extremes of inflammation and resolution. Our immunofluorescence data suggest that geraniin effectively harnesses this inherent plasticity, potentially facilitating the continuous interconversion of the macrophage population away from the tissue-destructive M1 pole and toward the mucosal-healing M2 pole. Given that an imbalance in the population of intestinal macrophages can transform the inflammatory landscape, orchestrating the equilibrium between M1 and M2 has lately been heralded as a promising therapeutic stratagem for ulcerative colitis (14). The polarization of macrophages is governed by a constellation of factors, with the activation of PPARG serving as a pivotal fulcrum in steering polarization toward the M2 phenotype (15, 16). Scholarly investigations affirm that PPARG is ubiquitously expressed within the monocyte–macrophage lineage, and its activation directly propels cells toward the M2 archetype. In instances where PPARG is rendered inactive, even amidst stimulation by interleukin-4 (IL-4), macrophages falter in their transition to the anti-inflammatory M2 phenotype (17). Evidence further demonstrates that PPARG, through its synergistic binding with other transcription factors to the promoter regions of genes such as Arg1, Fizz1, and Ym1, amplifies the expression of Arg1 and Fizz1, thereby modulating the degree of M2 polarization. In macrophages bereft of PPARG, the expression of Arg1 is notably diminished (18, 19). Nitric oxide (NO) operates dually as a downstream effector within the inflammatory cascade and as a critical upstream regulator. NO engages with an array of inflammatory mediators, participating in the signal transduction of diverse inflammatory pathways. Its production permeates all phases of the inflammatory response, rendering the bodily levels of NO a gage for the anti-inflammatory potency of pharmacotherapies. Pathologically excessive NO production is precipitated by NOS2, with stimuli such as bacteria, viruses, and inflammatory cytokines—including TNF-α and IL-6—capable of galvanizing NOS2 (20). M1 macrophages excel in generating toxic NO, augmenting NOS2 activity, and unleashing pro-inflammatory cytokines such as TNF-α, IL-6, and IL-1β, thereby intensifying the inflammatory maelstrom and perpetuating a deleterious cycle (21). The findings of this study suggest that in DSS-treated mice, the administration of geraniin is associated with an upregulation of PPARG protein expression and a concurrent downregulation of NOS2 protein expression, suggesting that geraniin may exert potential effects by modulating PPARG signaling to suppress M1 markers whilst fostering M2 markers.

Neutrophils, as indispensable constituents of the innate immune armamentarium within the intestinal mucosa, represent the vanguard of immune cells recruited to inflammatory loci, where they fulfill essential antimicrobial and immunoregulatory roles. In the context of UC, histopathological evidence consistently points to a pronounced elevation in neutrophil infiltration within the intestinal lining (22). This influx signifies a breach in intestinal homeostasis and is increasingly recognized as a central element in the pathophysiology of UC. Neutrophil extracellular traps (NETs), first characterized in 2004, constitute elaborate extracellular webs woven from decondensed chromatin fibers, richly decorated with histones and an array of potent granular proteins (23). While neutrophils leverage NET deployment, alongside the generation of reactive oxygen species and the release of proteolytic enzymes, to bolster their host defense capabilities, the overzealous or dysregulated formation of NETs (NETosis) can precipitate significant collateral injury to adjacent host tissues. Within the UC landscape, an ongoing debate surrounds the “paradox of NET involvement,” questioning whether they are merely bystanders of severe inflammation or active pathogenic drivers. Recent consensus suggests a highly context-dependent role: while baseline NET formation supports antimicrobial defense and tissue repair, their overzealous accumulation actively drives epithelial barrier disruption, bacterial translocation, and sustained mucosal inflammation, thus transitioning from a broad protective mechanism to an active contributor of disease progression (24, 25, 26). Substantial research now implicates aberrant NETosis in the etiology and perpetuation of diverse autoimmune and inflammatory conditions, including systemic lupus erythematosus, psoriasis, rheumatoid arthritis, and pertinently, UC (27, 28, 29, 30). Indeed, colonic biopsies procured from UC patients frequently exhibit markedly increased levels of NET-associated proteins. The REDD1/NETs/IL-1β signaling axis has emerged as a critical contributor to UC pathogenesis (31), and NETs have been shown to fuel UC-associated inflammation partly through mechanisms involving tumor necrosis factor-alpha (TNF-α) (32, 33). Furthermore, NETs can compromise the integrity of the intestinal epithelial barrier, thereby facilitating bacterial translocation and exacerbating gut inflammation. Congruent with this background, the present investigation revealed that geraniin administration in the DSS-induced colitis model significantly attenuated neutrophil migration to the inflamed colonic sites. Moreover, geraniin administration correlated with a reduced presence of the immunofluorescence markers associated with the excessive formation of NETs. While our immunofluorescence data indicated a reduction in NET formation, we acknowledge that this observation is currently limited to phenotypic marker expression. A detailed mechanistic dissection of the underlying signaling cascades, such as PAD4 activity or ROS-dependent pathways, falls beyond the scope of this initial study and will be systematically addressed in our future investigations.

Despite promising findings, this study has limitations. As an early preclinical exploration, direct validation in human UC cohorts is currently precluded by stringent ethical and toxicological prerequisites. However, our foundational targets were derived from human transcriptomic databases, supporting the potential clinical relevance of these observations. Furthermore, while our *in vivo* data show significant correlative modulation of inflammatory markers, future studies employing specific antagonists or conditional knockout models are warranted to further investigate mechanistic causality beyond these preliminary associations.

## 5 Conclusion

In conclusion, our preclinical findings suggest that the potential effects of geraniin in alleviating UC-like symptoms in mice likely correlate with a convergence of multiple immunomodulatory associations. Geraniin administration was found to be associated with a decrease in markers for M1 macrophages and a concurrent increase in M2 macrophages, potentially linked to the modulation of targets including PPARG and NOS2, alongside a reduction in phenotypic markers of excessive neutrophil recruitment and NET formation. Collectively, these preclinical observations suggest a concerted mechanism that may contribute to the significant dampening of the intestinal inflammatory response observed within this experimental model.

## Data Availability

The datasets generated and/or analyzed during the current study are available in the GEO (https://www.ncbi.nlm.nih.gov/, GSE47908,GSE214695). Full uncropped gels and blots images are provided as Supplementary material.

## Footnotes

1 Accessible at http://www.ncbi.nlm.nih.gov/geo.

2
https://satijalab.org/seurat
