# Supplementary material for: A preclinical investigation into the potential associations of geraniin with ulcerative colitis alleviation through integrated multi-omics and in vivo analysis
Source: Front Med (Lausanne). 2026 May 13;13:1821762. doi: 10.3389/fmed.2026.1821762 (PMC13212046; doi:10.3389/fmed.2026.1821762)
Supplement: Supplementary file 1 [file Supplementary_File_1.docx]

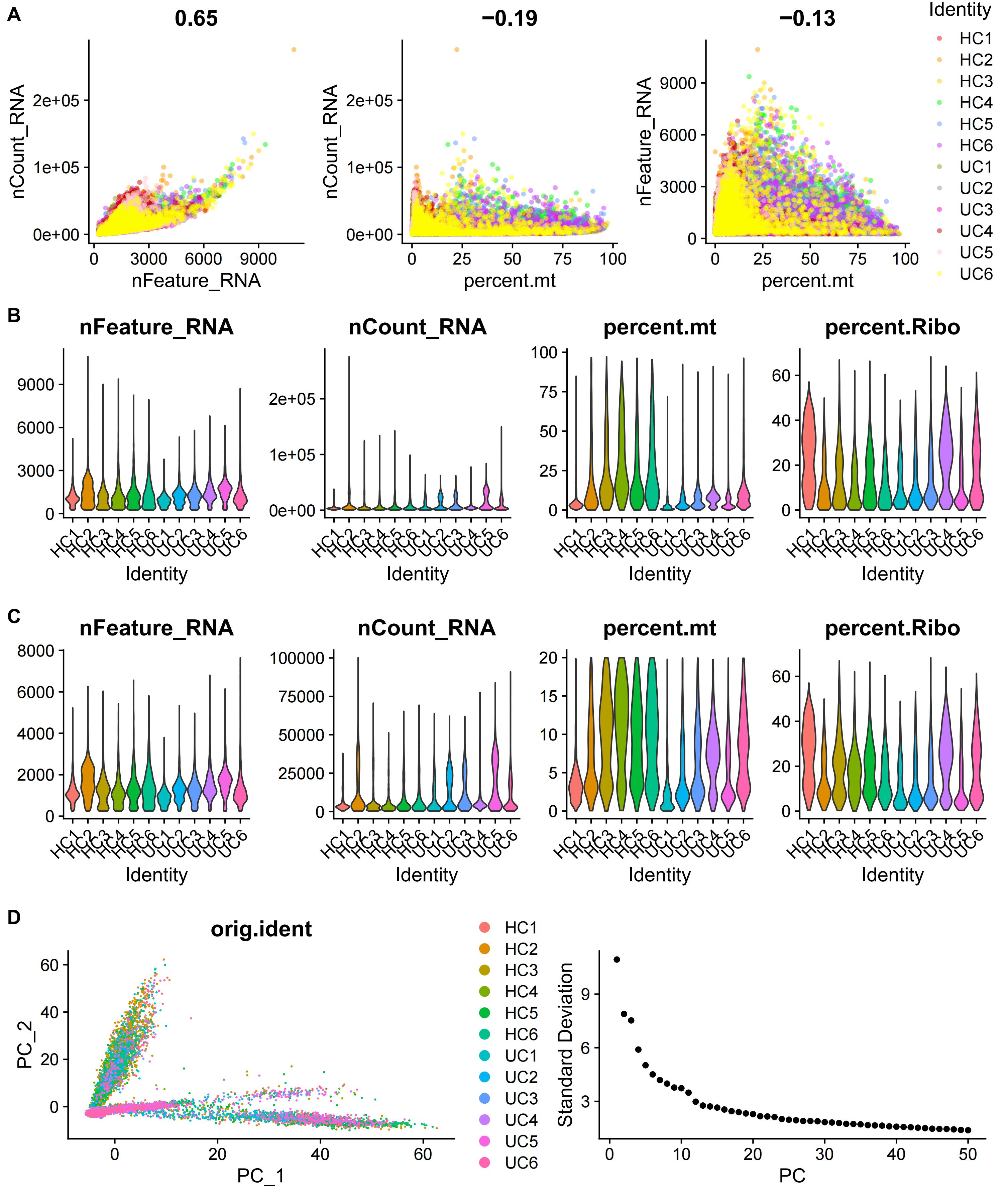


**Supplementary Figure S1. Quality control, data filtration, and dimensionality reduction of single-cell RNA sequencing data.** (A) Scatter plots demonstrating the correlations between the number of detected genes per cell (nFeature_RNA), total gene expression counts (nCount_RNA),and the proportion of mitochondrial genes (percent.mt) across 12 samples.Numbers at the top indicate the Pearson correlation coefficients. (B, C) Violin plots displaying the distributions of key quality control metrics (nFeature_RNA, nCount_RNA, percent.mt, and percent.Ribo) for each sample (B) before and (C) after the application of rigorous quality control criteria. Low-quality cells were filtered out by retaining only those with nFeature_RNA between 250 and 8,000, and percent.mt < 20%. (D) PCA plot showing the integration of samples colored by original identity (left), accompanied by an elbow plot (right) illustrating the standard deviation of each PC to determine the optimal dimensionality for subsequent clustering.


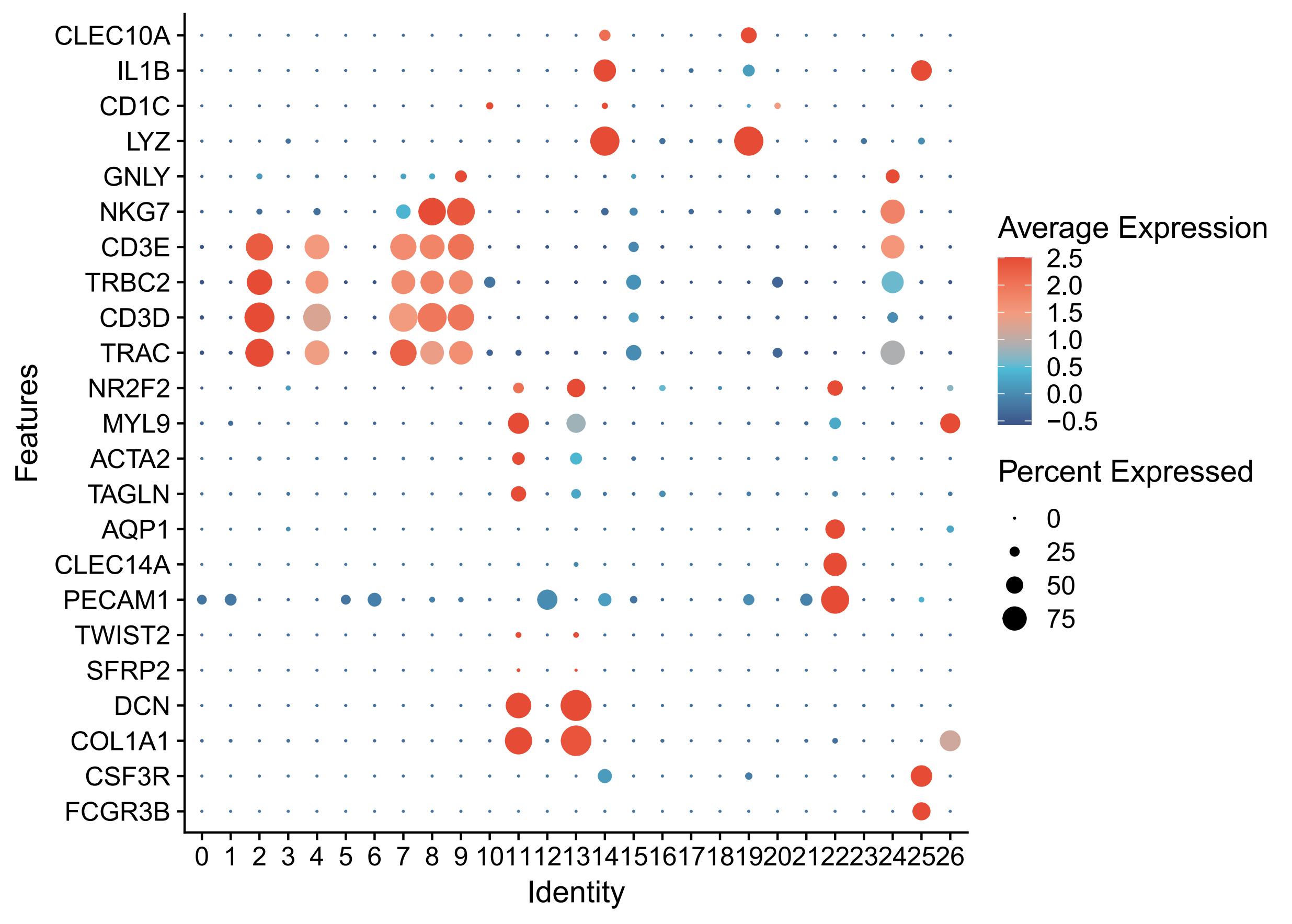


**Supplementary Figure S2. Expression profiles of canonical marker genes across identified single-cell subclusters.**
